# Integrative Multi‐Omics Analysis Reveals Rac GTPase Pathway Dysregulation Driving Lipid Metabolic Alterations in Vascular Cognitive Impairment

**DOI:** 10.1002/alz70856_101268

**Published:** 2025-12-24

**Authors:** Sangeetha Vishweswaraiah, Nadia Ashrafi, Romana Mimi, Nazia Saiyed, Abdullah Khalid, Ceyda Buyuker, Boran Aksakal, Lorena Gisele Ferreira Gomes, Milda Milčiūtė, Migle Gabrielaite, Ingrida Olendraite, Vilija Lomeikaite, Juozas Gordevičius, Ali Yilmaz, Stewart F Graham

**Affiliations:** ^1^ Corewell Health Research Institute, William Beaumont University Hospital, Oakland University‐William Beaumont School of Medicine, Royal Oak, MI, USA; ^2^ Corewell Health Research Institute, William Beaumont University Hospital, Royal Oak, MI, USA; ^3^ Corewell health research institue, Royal Oak, MI, USA; ^4^ Corewell Health University Hospital, Oakland University William Beaumont School of Medicine, Royal Oak, MI, USA; ^5^ Corewell health research institute, Royal Oak, MI, USA; ^6^ Acibadem Mehmet Ali Aydinlar University Ataşehir, Istanbul, Istanbul, Turkey; ^7^ VUGENE, Vilnius, Vilnius, Lithuania

## Abstract

**Background:**

Vascular cognitive impairment (VCI) encompasses a spectrum of cognitive disorders linked to vascular dysfunction, with underlying mechanisms remaining poorly understood. Traditional single‐omics approaches often fail to capture the complex biological networks driving VCI, necessitating integrative multi‐omics methodologies.

**Method:**

Brodmann area 7 brain tissue samples from 19 VCI cases and 21 controls were analyzed using a variety of “omics” platforms and were used to identify associations between metabolites, methylation, gene expression levels, and metabolic pathways associated with VCI.

**Result:**

Genomic analysis highlighted the Rac GTPase pathway in VCI pathogenesis, supported by widespread epigenetic alterations upregulating pathway‐associated genes. Further, transcriptomic data revealed dysregulation of lipid metabolism, oxidative stress, and GTPase activity, while our metabo‐epigenomics analyses highlighted disruption of diacylglycerol and phosphatidylethanolamine metabolism driven by Rac GTPase. This comprehensive approach identified association gains, losses, and reversals in disease states, providing a comprehensive understanding of VCI‐specific omics interactions.

**Conclusion:**

This study underscores the value of integrative multi‐omics in elucidating VCI mechanisms, revealing Rac GTPase‐driven lipid metabolic disruptions as a potential hallmark of the disease. These findings provide new insights into the molecular underpinnings of VCI, paving the way for biomarker development and targeted therapeutic strategies.

